# Society Meetings

**Published:** 1905-09

**Authors:** 


### SOCIETY MEETINGS.

The fifteenth International Medical Congress will be held in Lisbon, April 19-26, 1906. It is expected that this meeting will be of unusual importance, for one which will be held in what has always been considered as an out of the way country. Already the titles of papers from some of the most distinguished men of the medical profession have been received. Some of the topics for discussion that have been selected by the executive committee are the following:

*Section on Descriptive and Comparative Anatomy, Anthropology, Embryology and Histology.*—Definition, structure and composition of protoplasm; origin, nature and classification of pigments; cellular changes in normal tissues; evolution and involution of the thymus gland.

*Section on Physiology.*—The role of leucocytes in nutrition; the thyroid secretion; renal permeability; the nutritive value of alcohol; the physiology of the cytotoxins; the blood ferments.

*Section on General Pathology, Bacteriology and Pathological Anatomy.*—What are the present scientific proofs of the parasitic nature of neoplasms, especially of cancer? Preventive inocculation against bacterial diseases; preventive inoculations against protozoic diseases; preventive inoculations against diseases from an unknown specific agent; the pancreas and fat necrosis.

*Section on Therapeutics and Pharmacology.*—Local therapeutics in infectious diseases; separation, from a physiological and therapeutic point of view, of the different radiations produced in Crooke's tubes and of those which are sent out by radioactive bodies; the therapeutic value of bactericidal serums; the relation between the molecular constitution of organic bodies and their physiological and therapeutic action.

*Section on Medicine.*—The pathogenesis of diabetes; the pathogenesis of arterial hypertension; the treatment of cirrhosis of the liver; cerebrospinal meningitis; international defense against tuberculosis; meningeal hemorrhages.

*Section on Pediatrics.*—Spastic affections of infancy, classification and pathogenesis; cerebrospinal meningitis; etiology and treatment; the social struggle against rickets; orthopedic surgery in affections of nervous origin, spastic and paralytic; congenital dislocation of the hip; the treatment of abdominal tuberculosis (peritoneal).

*Section on Neurology, Psychiatry and Criminal Anthropology.*—Penal reform from the anthropologic and psychiatric point of view; forms and pathogenesis of dementia praecox; the relations of progressive muscular atrophy to Charcot's disease; cerebral localisation in mental disease; education and crime; stigmata of degeneration and crime.

*Section on Surgery.*—Septic peritoneal infections; classification and treatment; gastrointestinal and intestinointestinal anastomoses; recent additions to arterial and venous surgery.

*Section on Medicine and Surgery of the Urinary Organs.*—Surgical intervention in Bright's disease; surgical treatment of prostatovesical tuberculosis; progress of urology in the diagnosis of renal disease; painful cystides.

*Section on Ophthalmology.*—Blepharoplasty; serotherapy in ophthalmology.

*Section on Laryngology, Rhinology, Otology and Stomatology.*—Study of the epileptogenous action of foreign bodies in the ear and of vegetations in the nasopharynx; the different forms of suppuration of the maxillary sinus; injections of paraffin in rhinology; differential diagnosis of tubercular, syphilitic and cancerous lesions of the larynx; choice of anesthesia in the extraction of teeth; treatment of alveolar suppuration.

*Section on Obstetrics and Gynecology.*—Conservative surgery of the ovaries; tuberculosis of the adnexa; symphyseotomy; pregnancy and cancer of the uterus; therapy of puerperal infections.

*Section on Hygiene and Epidemiology.*—The intermediary of yellow fever; the coöperation of nations to prevent the importation of yellow fever and the pest; watering the streets as a means against tuberculosis; recent additions to the etiology and epidemiology of epidemic cerebrospinal meningitis.

*Section on Military Medicine.*—Portable ration of the soldier during campaign; the purifying of the country water; emergency hospitals on the battlefield.

*Section on Legal Medicine.*—Signs of death from drowning; ecchymoses in legal medicine; epilepsy in legal medicine; organisation of medicolegal services.

*Section on Colonial and Naval Medicine.*—Etiology and prophylaxis of beri-beri; etiology and prophylaxis of dysentery in hot countries; mental diseases in tropical countries; hospital ships and their function in time of war; tuberculosis in the navy and its prophylaxis.

---

The Gross Medical Club was entertained by Dr. W. A. Macpherson, at Leroy, during its regular monthly meeting held August 18, 1905. The meeting was attended by Drs. J. Henry Dowd, R. H. Satterlee, C. O. Chester, and B. H. Grove, of Buffalo. Dr. Beach, of Corfu, read the paper of the occasion.

---

The Ohio Society of United States Pension Examining Surgeons, recently organised, held its first annual meeting May 11, 1905, at Columbus.

---

The Medical Association of Central New York will hold its thirty-eighth annual meeting at Buffalo, Tuesday, October 17, 1905, under the presidency of Dr. Charles G. Stockton of this city. Dr. William C. Krauss is the chairman of the committee of arrangements.

---

The Medical Society of the County of Wayne at its recent annual meeting elected the following-named officers: president, Fred L. Wilson, Sodus Point; vice-president, Lacey B. Darling, Palmyra; secretary, Andrew F. Sheldon, Lyons; treasurer, M. A. Veeder, Lyons.

---

A joint meeting of the medical societies of the counties of Cattaraugus (N. Y.) and of McKean (Pa.) was held at Rock City, August 8 and 9, 1905. The attendance included physicians from Buffalo, Philadelphia, and Pittsburg. Among those present from Buffalo, were: Drs. Henry C. Buswell, Matthew D. Mann, E. J. Meyer, W. C. Krauss, W. W. Plummer, and Roswell Park.

---

The American Academy of Ophthalmology and Otolaryngology will hold its tenth annual meeting at the Lenox Hotel, Buffalo,

September 14-16, 1905, under the presidency of Dr. Hanau W. Loeb, of Saint Louis. Dr. Alvin A. Hubbell is chairman of the committee on arrangements, and Drs. F. Park Lewis, A. A. Hubbell, and Lucien Howe, of this city, are scheduled to participate in the scientific proceedings.

